# Characterization of Coronary Artery Disease in Sepsis Survivors

**DOI:** 10.3390/biomedicines13051181

**Published:** 2025-05-13

**Authors:** Samuel Malomo, Thomas Oswald, Thomas Alway, Stanislav Hadjivassilev, Steven Coombs, Susan Ellery, Joon Lee, Claire Phillips, Barbara Philips, Rachael James, David Hildick-Smith, Victoria Parish, Alexander Liu

**Affiliations:** 1 Sussex Cardiac Centre, Royal Sussex County Hospital, Brighton BN2 5BE, UK; s.malomo@nhs.net (S.M.); thomas.oswald@nhs.net (T.O.); t.alway@nhs.net (T.A.); stanislav.hadjivassilev@nhs.net (S.H.); steven.coombs@nhs.net (S.C.); susan.ellery1@nhs.net (S.E.); joon.lee@nhs.net (J.L.); rachael.james3@nhs.net (R.J.); david.hildick-smith@nhs.net (D.H.-S.); victoria.parish@nhs.net (V.P.); 2 Intensive Care Unit, Royal Sussex County Hospital, Brighton BN2 5BE, UK; claire.phillips20@nhs.net (C.P.); b.philips@bsms.ac.uk (B.P.); 3 Brighton and Sussex Medical School, Brighton BN1 9PX, UK

**Keywords:** sepsis, coronary artery disease, ischemic heart failure, cardiovascular computed tomography, heart failure

## Abstract

**Background:** Sepsis survivors are at risk of developing myocardial infarction and heart failure. It remains unclear whether coronary artery disease (CAD) is a major contributor to the development of these complications. This study sought to characterize the burden and distribution of significant CAD in sepsis survivors. **Methods:** Sepsis survivors who underwent computed tomography coronary angiography (CTCA) or invasive coronary angiography (ICA) in a UK tertiary cardiac center for suspected ischemic heart disease were retrospectively studied. **Results:** Of the 30 sepsis survivors (age 57 ± 12 years; 50% males), 21 patients underwent CTCA and 9 patients underwent ICA a median 39 days [IQR 12–152] from the sepsis episode. Eight patients (~27%) had angiographically significant CAD (n = 6 severe [>70%] stenosis; n = 2 moderate [50–70%] stenosis). The CT coronary calcium score was higher in patients with significant CAD compared to patients without significant CAD (638 [368–1015] vs. 4 [1–72]; *p* < 0.001). Of the 8 patients with significant CAD, 3 patients had LV systolic dysfunction (38%) on echocardiography and 8/21 (38%) patients without significant CAD had LV systolic dysfunction (*p* = 1.00). Long-term adverse complications (all-cause mortality and/or heart failure hospitalization) occurred 3/8 (38%) patients with significant CAD and 4/22 (18%) patients without significant CAD (*p* = 0.345). **Conclusions:** A minority of sepsis survivors have significant CAD. The presence of significant CAD cannot fully explain the occurrence of post-sepsis LV systolic dysfunction and adverse outcomes. The ischemic and non-ischemic mechanisms underlying post-sepsis cardiovascular disease require further investigation.

## 1. Introduction

Sepsis remains a leading cause of morbidity and mortality globally [1]. Each year, around 49 million cases of sepsis are recorded worldwide, leading to around 11 million deaths [1]. Sepsis survivors have an elevated risk of developing long-term cardiovascular complications, such as myocardial infarction and heart failure, even after recovery from the acute septic episode [2]. The pathophysiology of this elevated cardiovascular risk remains unclear [2], which limits the scope for novel and targeted therapeutic development.

Significant coronary artery disease (CAD) is a major conventional risk factor for the development of acute myocardial infarction and cardiac dysfunction [3,4]. The burden of significant CAD in sepsis survivors remains unknown, which may represent an important factor in the pathogenesis of the observed cardiac complications [2]. In patients with suspected ischemic heart disease, anatomically significant CAD can be detected by invasive coronary angiography, or non-invasively by computed tomography coronary angiography (CTCA) [5]. This study sought to characterize the burden and distribution of significant CAD in sepsis survivors in relation to cardiac dysfunction and long-term cardiovascular complications.

## 2. Materials and Methods

### 2.1. Study Subjects

Sepsis survivors over the age of 18 years who underwent CTCA or ICA between September 2014 and October 2024 at the Royal Sussex County Hospital, Brighton (a UK tertiary cardiac center) for suspected ischemic heart disease were included in the study. Patients were excluded if they did not proceed to CTCA due to high coronary artery calcium scores (n = 2) or had previously undergone coronary artery revascularization (n = 1). As a proof-of-concept study, formal power calculation was not performed. The study sample size (n = 30) was determined by the number of suitable patients in the clinical database available for inclusion. This sample size was on a similar scale to other proof-of-concept studies in sepsis patients [6,7].

### 2.2. Ethical Approval

This retrospective study was approved by the Research and Innovation department of the University Hospitals Sussex NHS Foundation Trust, and informed patient consent was waived.

### 2.3. Clinical Data Collection

Clinical parameters of the study’s patients were collected from the electronic medical records. These included demographics data, acute sepsis data, cardiac symptoms, co-morbidities and regular cardiac medications. Clinical outcomes data were also collected from the electronic medical records. Long-term adverse complications were defined as a composite of all-cause mortality, myocardial infarction, stroke and heart failure hospitalization. The data collected were independently validated by a second observer as referenced to the electronic medical records.

### 2.4. CTCA

CTCA scans were performed using a 320-row detector scanner (Aquilion ONE PRISM, Canon Medical Systems Corporation, Otawara, Japan). Non-contrast images with slice thicknesses of 3 mm were first acquired for coronary artery calcium scoring using a 120 kVp prospective ECG-gated protocol. Contrast-enhancement coronary angiography was then performed with slice thicknesses of 0.5 mm using a 120 kVp prospective ECG-gated protocol. The CT scan images were analyzed by experienced clinical cardiology or radiology consultant physicians using commercially available software (Syngo.via, version VB60A, Siemens Healthineers, Forchheim, Germany). The scans were independently verified by another consultant physician as part of the study data analysis. Coronary artery calcium scoring was performed using the Agatston method [8]. Significant coronary artery disease was defined as either moderate (50–70% stenosis) or severe (>70% stenosis) by the CAD-RADS classification [9].

### 2.5. ICA

Invasive coronary angiography was performed as part of the patients’ clinical care by experienced consultant cardiologists. Images were acquired in multiple orthogonal planes. Similar to CTCA assessment, significant CAD was defined as either moderate (50–70% stenosis) or severe (>70% stenosis) by visual assessment.

### 2.6. Transthoracic Echocardiography (TTE)

Standard 2D TTE images, color flow and Doppler images were acquired using commercially available systems in parasternal, apical and sub-costal views over 3 cardiac cycles. Image acquisition was optimized by adjusting the sector size, depth and gain settings. LV systolic function was assessed visually or by the Simpson biplane method.

### 2.7. Statistical Analysis

Parametric data were displayed as mean ± standard deviation. Non-parametric data were displayed as median [interquartile range]. Continuous variables were compared using the Mann–Whitney test. Categorical variables were compared using Fisher’s exact test. *p* values < 0.05 denote statistical significance. Statistical analysis was performed using commercially available software (MedCalc, version 20.104, Mariakerke, Belgium). All data analyses and results were validated by a second observer for accuracy.

## 3. Results

### 3.1. Clinical Data of Sepsis Survivors

Of the 30 sepsis survivors (57 ± 12 years; 50% males), pneumonia was the commonest cause of sepsis (40% Table 1): 14 patients (47%) required intensive care unit (ICU) admissions; 4 patients (13%) required management on the high-dependency unit (HDU) or the cardiac care unit (CCU); and 12 patients (40%) were treated on the medical wards (Table 1). During acute sepsis, patients had elevated peak C-reactive protein levels (CRP; 244 [71–316] mg/L), white cell counts (16.5 × 10^9^/L [IQR: 12.0–22.9]) and high sensitivity cardiac troponin T (hs-cTnT) levels (108 ng/L [16–785]).

The cardiac symptoms, co-morbidities and medications of sepsis survivors are shown in Table 2. Most of the patients had at least one cardiac symptom, such as chest pain (47%), dyspnea (33%), palpitations (13%) and/or presyncope/syncope (3%), which contributed to the triggers for their coronary artery assessment.

### 3.2. Distribution of CAD in Sepsis Survivors

Of the 30 sepsis survivors, 21 patients underwent CTCA and 9 patients underwent ICA a median 39 days [IQR 12–152] from the sepsis episode.

On a per-patient basis, 8 out of the total 30 patients (27%) had significant CAD. Of the 8 patients with significant CAD, 6 patients had at least one severe stenosis (angiographically >70%) and 2 patients had at least one moderate stenosis (angiographically 50–70%). Four patients had multivessel significant CAD.

On a per-vessel basis, 14 out of a total of 120 major coronary arteries (LMS, LAD, RCA, LCx) had significant CAD. Of these 14 vessels, 8 vessels had severe stenosis (n = 4 LAD; n = 1 LCx; n = 4 RCA; Table 3). The remaining six vessels with significant CAD had moderate stenosis (n = 2 LAD; n = 2 LCx; n = 2 RCA; Table 3). There was no LMS involvement (defined as >50% stenosis).

CT coronary artery calcium scores were higher in patients with significant CAD (638 [368–1015]) compared to patients without significant CAD (4 [1–72]), *p* < 0.001.

Of the eight patients with significant CAD, two patients received percutaneous coronary intervention with medical therapy, and six patients were managed with medical therapy alone.

### 3.3. Relationship Between CAD and TTE/Outcomes Data in Sepsis Survivors

Of the 30 study patients, 11 (37%) patients had LV systolic dysfunction (LVEF < 50%) on TTE (Table 3). Of the 8 patients with significant CAD, 3 patients had LV systolic dysfunction (38%) and 8/21 (38%) patients without significant CAD had LV systolic dysfunction (*p* = 1.00; Table 3). Over a follow-up period of 16 months [2–40], long-term adverse complications (n = 5 all-cause mortality; n = 2 heart failure hospitalizations) occurred in 3/8 (38%) patients with significant CAD and 4/22 (18%) patients without significant CAD (*p* = 0.345; Table 3).

**Figure 1:**
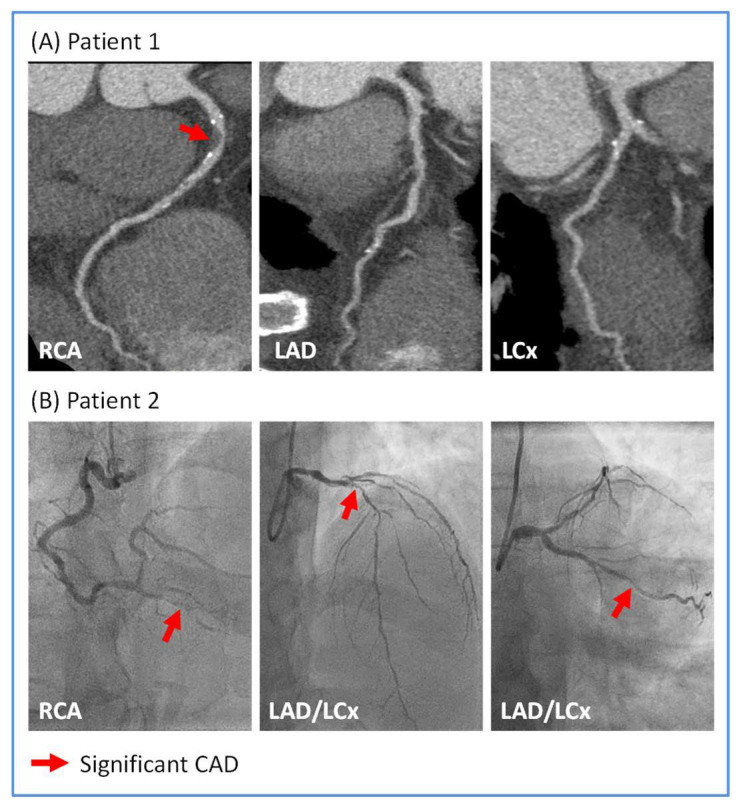
Illustrative images of significant coronary artery disease (CAD) in sepsis survivors. (**A**) Patient 1 underwent computed tomography coronary angiography which showed severe (>70%) stenosis in the proximal right coronary artery (RCA; red arrow) and unobstructed left anterior descending (LAD) and left circumflex (LCx) arteries. (**B**) Patient 2 underwent invasive coronary angiography which showed severe stenoses in the distal RCA, the proximal LAD and mid LCx arteries (all indicated by red arrows).

Illustrative examples of CTCA and ICA of sepsis survivors are shown in Figure 1.

## 4. Discussion

This study evaluated the presence and distribution of significant CAD in a cohort of sepsis survivors. The main findings were as follows: (i) a minority of sepsis survivors suspected of having ischemic heart disease actually have angiographically significant CAD (27%); (ii) a minority of the sepsis survivors with angiographically significant CAD underwent PCI, and most cases were managed with medical therapy alone; (iii) the frequency of LV systolic dysfunction was similar in patients with significant CAD vs. patients without significant CAD; and (iv) the frequency of long-term adverse complications was also similar in patients with significant CAD vs. patients without significant CAD.

These findings do not appear to support the major role played by significant CAD in the development of post-sepsis LV systolic dysfunction and composite cardiovascular outcomes. A larger study, ideally performed on a multi-center basis, is required to confirm these proof-to-concept findings. If confirmed, it would provide further evidence in support of a non-ischemic mechanism underlying the development of post-sepsis cardiomyopathy.

### 4.1. The Post-Sepsis Cardiovascular Risk

Recent observational data have highlighted that sepsis survivors have an elevated long-term risk of developing myocardial infarction, strokes, heart failure and mortality [2,10,11,12]. These data appear to challenge the traditional paradigm that sepsis related cardiac dysfunction and ischemic risk reverse after recovery from sepsis [13,14]. Studies have also shown that increased levels of coronary artery calcification have a prognostic impact on sepsis survivors [15,16]. Further, post-sepsis patients with coronary artery calcification and LV systolic dysfunction appear to have a high risk of developing cardiovascular complications [16]. However, coronary artery calcification does not accurately reflect the degree of coronary luminal stenosis, which is a stronger predictor of future ischemic events [3,9].

The present study showed that only a minority of sepsis survivors suspected of having ischemic heart disease, e.g., presenting with chest pains or LV dysfunction, actually had significant CAD. In fact, the majority of these patients had normal or unobstructed coronary arteries. Moreover, the frequence of LV systolic dysfunction was similar between sepsis survivors with and without significant CAD. This suggests that post-sepsis LV dysfunction cannot be explained by the presence of coronary disease alone, rather, a non-ischemic mechanism may also play an important role in its pathophysiology. Contemporary studies showed that acute sepsis patients develop significant myocardial oedema and stress-related LV dysfunction, which are linked to myocardial injury [6,17]. Some of these changes are similar to those observed in other inflammatory cardiomyopathies associated with cardiac dysfunction [18,19,20] and, more recently, in coronavirus disease 2019 patients who suffer cardiac injury [21,22,23,24,25]. Whether the myocardial tissue abnormalities observed during acute sepsis persist or progress in the post-sepsis period remains unclear, primarily owing to the lack of serial cardiac imaging studies. This is an area of important future research.

In this study, the frequency of adverse complications was similar between sepsis survivors with and without significant CAD. The significance of this observation is three-fold. Firstly, it may reflect that factors other than significant CAD and myocardial ischemia may exert a stronger influence on the likelihood of sepsis survivors developing long-term cardiac complications. Secondly, the prevalence of significant CAD in this patient cohort may be too low to become the major cause of cardiac complications. Thirdly, the primary endpoint in the study was limited to all-cause mortality and heart failure hospitalization (no myocardial infarction or stroke occurred), which may have skewed the outcome status of significant CAD in the sepsis survivors. Despite the observed low frequency of significant CAD in sepsis survivors, it remains clinically important to perform coronary assessments in these patients with active symptoms or LV dysfunction, since some patients did indeed demonstrate severe CAD, including multi-vessel disease. A further larger study is needed to test the validity of the observations in this study.

### 4.2. Limitations and Future Directions

This retrospective study had a relatively small sample size, which means that the results are prone to sampling bias. The results will be validated in a larger study, which includes multi-modality cardiac imaging. Patients were referred for coronary artery assessments on the grounds of having suspected ischemic heart disease and/or cardiac symptoms, which may not be representative of the broader sepsis survivor population. A larger population-based study would further address this limitation and assess the burden of asymptomatic coronary artery disease in post-sepsis patients. Most patients did not undergo cardiovascular magnetic resonance (CMR) imaging which would have provided further data on their myocardial structure and tissue characterization [7,26,27]. This is the topic of a separate study which will yield further insights into the cardiomyopathic phenotype of sepsis survivors. No myocardial infarction or stroke occurred in the study patients, and the composite outcome was driven mainly by mortality and heart failure hospitalization. All-cause mortality was recorded, and cardiac mortality was not specifically adjudicated. These limitations of the clinical outcomes should be addressed in a larger prospective study. Although angiographic classifications were applied in accordance with established criteria for CAD severity, most patients did not undergo coronary artery physiological studies such as fractional flow reserve (FFR). These measurements would have provided further information on the physiological significance of the coronary stenoses, in particular for moderate lesions known to have prognostic value overall [28]. By the same token, the limited use of non-invasive functional tests in patients with angiographically significant CAD would have also hindered the assessment of myocardial ischemia and its relationship with cardiac function and outcomes [29,30]. Future studies using multi-parametric assessment of invasive coronary studies and non-invasive functional tests would be important to further assess the physiological significance of coronary disease in post-sepsis patients [31,32,33,34,35]. The link between significant CAD and the precise aetiology of LV systolic dysfunction in post-sepsis patients remains unclear, which should be further investigated using multi-modality cardiac imaging [36,37,38]. Despite these limitations, this study is the first to characterize the burden and distribution of coronary artery disease in sepsis survivors.

## 5. Conclusions

A minority of sepsis survivors have significant CAD. The presence of significant CAD cannot fully explain the occurrence of post-sepsis LV systolic dysfunction and adverse outcomes. The ischemic and non-ischemic mechanisms underlying post-sepsis cardiovascular disease require further investigation.

## Figures and Tables

**Table 1:** Clinical features of the sepsis event in patients.

	Patients (n = 30)
Age, years	57 ± 12
Male	15 (50)
BMI, kg/m^2^	29 ± 5
Sepsis cause	
Pneumonia	12 (40)
Unknown origin	4 (13)
Endocarditis	5 (17)
Abscess/Soft tissue	4 (13)
Gastrointestinal	2 (7)
Cellulitis	2 (7)
Urinary tract infection	1 (3)
Care escalation	
ICU	14 (47)
HDU/CCU	4 (13)
Ward-based care	12 (40)
Support requirements	
Intubation	5 (17)
Vasopressor	5 (17)
Inotrope	4 (13)
Serum biomarkers	
Peak CRP, mg/L	244 [71–316]
Peak WCC, ×10^9^/L	16.5 [12.0–22.9]
Peak Hs-cTnT, ng/L	108 [16–785]

CCU: cardiac care unit; CRP: C-reactive protein; HDU: high dependency unit; Hs-cTnT: high sensitivity cardiac troponin T; ICU: intensive care unit; and WCC: white cell count. Continuous variables are displayed as mean ± SD or median [interquartile range]. Categorical variables are displayed as number (%).

**Table 2:** Baseline clinical characteristics of post-sepsis patients and controls.

	Patients (n = 30)
Cardiac symptoms	
Chest pain	14 (47)
Dyspnoea	10 (33)
Palpitations	4 (13)
Pre-syncope/Syncope	1 (3)
Co-morbidities	
Atrial fibrillation	10 (33)
Hypertension	7 (23)
Diabetes mellitus	6 (20)
Smoking (ex- or current)	9 (30)
Hypercholesterolaemia	9 (30)
CKD	2 (7)
Medications	
Anti-platelet drugs	12 (40)
ACE-inhibitor/ARB	13 (43)
Beta-blocker	15 (50)
Sacubitril/Valsartan	3 (10)
MRA	8 (27)
SGLT-2 inhibitor	7 (23)
Calcium channel blockers	2 (7)
Loop diuretics	6 (20)
Statin	13 (43)
Anticoagulation	8 (27)

ACE: angiotensin converting enzyme; ARB: angiotensin receptor blocker; BMI: body mass index; BSA: body surface area; CKD: chronic kidney disease; COPD: chronic obstructive airways disease; CVA: cerebrovascular accident; MRA: mineralocorticoid receptor antagonist; SGLT-2: Sodium-glucose co-transporter-2; and TIA: transient ischemic attack. Continuous variables are displayed as mean ± SD or median [interquartile range]. Categorical variables are displayed as number (%).

**Table 3:** Coronary assessment, echocardiography and clinical outcomes data.

	Patients (n = 30)
Coronary artery assessment	
CTCA	21 (70)
ICA	9 (30)
Days from sepsis episode	39 [12–152]
Per-patient CAD (one or more of the following)	
Severe stenosis (>70%)	6 (23)
Moderate stenosis (50–70%)	2 (7)
Per-vessel CAD	
Severe stenosis (>70%)	
Total	8 (27)
LMS	0 (0)
LAD	4 (13)
LCx	1 (3)
RCA	3 (10)
Moderate stenosis (50–70%)	
Total	6 (20)
LMS	0 (0)
LAD	2 (7)
LCx	2 (7)
RCA	2 (7)
Multi-vessel disease †	4 (13)
CT coronary artery calcium score	
CAD positive (≥50% stenosis)	638 [368–1015]
CAD negative (<50% stenosis)	4 [1–72]
Intervention	
PCI and medical therapy	2 (7)
Medical therapy alone	6 (23)
Echocardiography data	
LVEF ≥50%	19 (63)
LVEF <50%	11 (37)
LVIDd, cm	4.9 ± 0.8
LVIDs, cm	3.1 [2.6–3.5]
Septal wall thickness, cm	1.0 [0.9–1.2]
Posterior wall thickness, cm	1.1 [0.9–1.2]
TAPSE, cm	2.1 ± 0.4
RV S’	13 ± 4
LA volume, cm^2^	48 [40–72]
Clinical outcomes data	
Follow up period, months	16 [2–40]
Total composite end points	7 (23)
Death	5 (17)
Heart failure hospitalization	2 (7)

† One or more vessels with moderate or severe coronary stenosis. CTCA: computed tomography coronary angiogram; EF: ejection fraction; ICA: invasive coronary angiogram; LA: left atrium; LAD: left anterior descending; LCx: left circumflex; LMS: left main stem; LV: left ventricular; LVIDd: LV internal diameter at end diastole; LVIDs: LV internal diameter at end systole; PCI: percutaneous coronary intervention; RCA: right coronary artery; RV: right ventricular; and TAPSE: Tricuspid annular plane systolic excursion.

## Data Availability

Patient clinical data in the study cannot be publicly shared but anonymized versions can be provided on reasonable request to the corresponding author.
